# Extracellular matrix cues regulate the differentiation of pluripotent stem cell-derived endothelial cells

**DOI:** 10.3389/fcvm.2023.1169331

**Published:** 2023-06-26

**Authors:** Kyung Mu Noh, Soon-Jung Park, Sung-Hwan Moon, Seok Yun Jung

**Affiliations:** ^1^ Stem Cell Research Institute, T&R Biofab Co. Ltd., Seongnam-si, Republic of Korea; ^2^ Department of Animal Science and Technology, College of Biotechnology and Natural Resources, Chung-Ang University, Anseong-si, Republic of Korea

**Keywords:** stem cell, induced pluripotent stem cell, endothelial cell, mechanical cues, extracellular matrix

## Abstract

The generation of endothelial cells (ECs) from human pluripotent stem cells (PSCs) has been a promising approach for treating cardiovascular diseases for several years. Human PSCs, particularly induced pluripotent stem cells (iPSCs), are an attractive source of ECs for cell therapy. Although there is a diversity of methods for endothelial cell differentiation using biochemical factors, such as small molecules and cytokines, the efficiency of EC production varies depending on the type and dose of biochemical factors. Moreover, the protocols in which most EC differentiation studies have been performed were in very unphysiological conditions that do not reflect the microenvironment of native tissue. The microenvironment surrounding stem cells exerts variable biochemical and biomechanical stimuli that can affect stem cell differentiation and behavior. The stiffness and components of the extracellular microenvironment are critical inducers of stem cell behavior and fate specification by sensing the extracellular matrix (ECM) cues, adjusting the cytoskeleton tension, and delivering external signals to the nucleus. Differentiation of stem cells into ECs using a cocktail of biochemical factors has been performed for decades. However, the effects of mechanical stimuli on endothelial cell differentiation remain poorly understood. This review provides an overview of the methods used to differentiate ECs from stem cells by chemical and mechanical stimuli. We also propose the possibility of a novel EC differentiation strategy using a synthetic and natural extracellular matrix.

## 1. Introduction

Endothelial cells (ECs) are compacted epithelial cells located on the inner surface of blood and lymphatic vessels. The primary function of these cells is to exchange molecular components between the circulating blood and the underlying connective tissue. The most prevalent ECs in the human vasculature are mature ECs that strongly express the pan-endothelial markers CD31 and CD144 (VE-cadherin) on their surface.

Cardiovascular diseases are a leading cause of death worldwide, with limited options to restore healthy blood vessels (1). Dysfunctional ECs are the primary cause of cardiovascular diseases, including hypertension and coronary artery disease (2). To treat dysfunctional ECs, research into the production of stem cell-derived vascular endothelial cells is of considerable interest in regenerative medicine.

Embryonic stem cells (ESCs) are pluripotent stem cells (PSCs) with an unlimited self-renewal capacity derived from the inner cell mass of blastocyst-stage embryos. ESCs generally remain undifferentiated under defined culture conditions but can differentiate into three germline lineages when exposed to specific stimuli. Notably, induced PSCs (iPSCs) have a high degree of similarity to ESCs in terms of development and morphology, as well as the pluripotency to differentiate into all three germlines in response to stimuli (3, 4). iPSCs were first described by Yamanaka et al. as a unique cell type derived from somatic cell reprogramming with the overexpression of exogenous transcription factors (Yamanaka factors: Oct4, Sox2, Klf4, and c-Myc) (5). These reprogrammed iPSCs can be obtained from various tissue cell types, including hepatocytes, peripheral blood mononuclear cells, cord blood cells, gastric epithelial cells, fibroblasts, urine cells, and keratinocytes (6–8). Unlike ESCs, iPSCs have no ethical problems and can be produced on a large scale, so they are considered an attractive source for developing cell therapy products in regenerative medicine (9). Therefore, the differentiation of PSCs into ECs is gaining increasing attention because it offers the opportunity to restore the blood vessels and can be used to cardiovascular disease.

Recently, studies on endothelial cell differentiation based on various small molecules and cytokines have been performed using extracellular matrix (ECM) cues (10–12). The microenvironment in which the cells responsible for tissue function exist in the human body is called the ECM. These cells are influenced by mechanical cues from the ECM environment, such as substrate stiffness, ECM components, and topological effects (13). Living cells can sense mechanical signals from the ECM environment surrounding the cells and convert them into biochemical signals that lead to functional changes such as cell morphology, cell migration, proliferation, and differentiation, which is referred to as mechanotransduction (14). Adam Engler et al. have investigated the differentiation fate of stem cells through ECM stiffness. They have demonstrated that the differentiation of stem cells into each specific cell type was promoted in a substrate that mimics the stiffness of specific tissue environments, such as neuronal (0.1–1 kPa), muscle (8–17 kPa), and bone (25–40 kPa) (14, 15). Although substrate stiffness is a vital parameter in determining stem cell differentiation fate, most studies on stem cell-derived endothelial differentiation have been performed with 2D- or 3D-based methods using biochemical factors. The 2D monolayer-based endothelial differentiation method uses ECM-coated tissue culture plates (TCP, approximately 10^6^ kPa) (16, 17), which showed that the microenvironment in which most cells are cultured *in vitro* does not reflect physiological stiffness value (18). The 3D embryoid body (EB) formation approach produces ECs with a low efficiency of 2%–20% and heterogeneous results (19–21). Therefore, it is vital to create a microenvironment that mimics the stiffness and ECM components of blood vessels to generate stem cell-derived ECs to treat cardiovascular disease.

Treating cardiovascular diseases using stem cell-derived ECs has been established as a promising therapeutic strategy. However, the role of mechanical cues from the ECM in governing EC differentiation remains poorly understood. In this review, we comprehensively described the existing knowledge on the effects of biochemical and mechanical stimuli on EC differentiation and proposed the possibility of a novel EC differentiation strategy using a synthetic and natural ECM. Investigating mechanical properties associated with the vascular microenvironment and their application to EC generation would help provide novel insights into the development of strategies and directions for treating cardiovascular diseases. Thus, this review will contribute to our mechanistic understanding of EC differentiation and serve as a foundation for further research on EC-based cardiovascular disease treatment.

## 2. PSC-derived EC differentiation regulated by biochemical factors

### 2.1. Mesoderm induction for endothelial lineage

**Figure 1:**
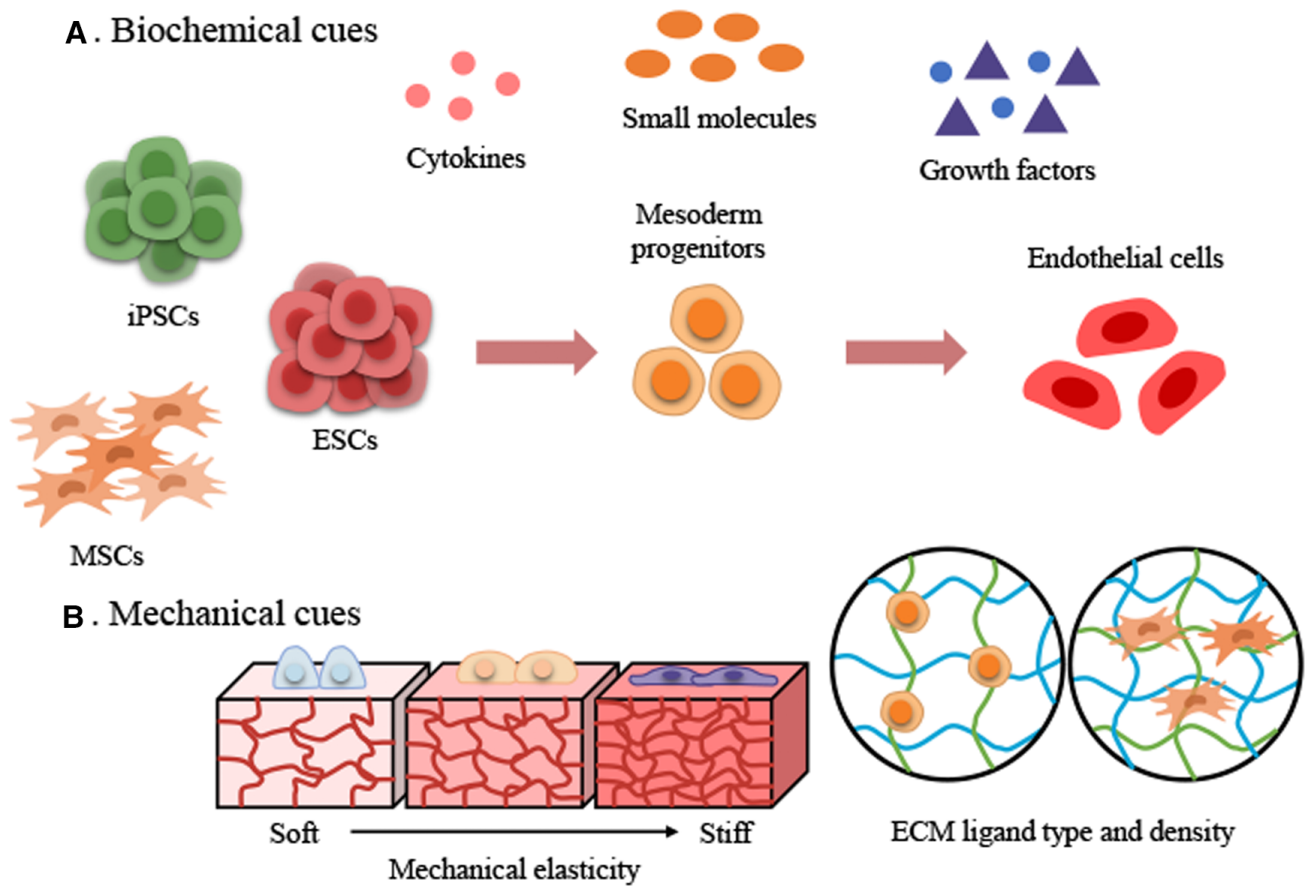
Two major approaches to achieve stem cell-derived endothelial cell differentiation using biochemical and mechanical cues. (**A**) Biochemical cues. (**B**) Mechanical cues. ECM, extracellular matrix; ESCs, embryonic stem cells; iPSCs, induced pluripotent stem cells; MSCs, mesenchymal stem cells.

During the development of mammalian embryos, ECs from the mesoderm first form the primitive vascular plexus, an immature tubular network, which matures into an orderly network of arteries, veins, and capillaries (22). The concept of differentiation of PSC-ECs is derived from vascular development in embryology. In recent decades, efforts have been made to efficiently differentiate ECs from PSCs under the influence of biochemical factors (23–29). These approaches are based on 2D and 3D EC differentiation protocols, in which the basal medium contains various cytokine supplements (Figure 1A). EC differentiation methods based on biochemical factors usually consist of several phases, such as mesoderm induction, vascular specification, and EC maturation (Table 1). First, different types and concentrations of small molecules and cytokines [CHIR99021, CP21R7, BMP4, Activin A, fibroblast growth factor 2 (FGF2)] were used to differentiate PSCs into mesodermal lineage cells. A glycogen synthase kinase-3 (GSK3β) chemical inhibitor (CHIR99021) was added to the cytokine cocktail for mesoderm induction to differentiate the mesoderm (31). However, inhibition of GSK3β could promote self-renewal or mesendoderm differentiation of human PSCs (hPSCs) (32–34). The mesoderm-inducing effects of GSK3 inhibitors, including CHIR99021, CP21R7 (CP21), 6-bromoindirubin-3′-oxime (BIO), and SB216763, were investigated to inhibit GSK3 and activate Wnt signaling. CP21 and CHIR99021, selected by a luciferase assay and cell viability assay, were used for mesoderm induction, and these mesodermal cells can differentiate into different types of vascular lineage cells, such as endothelial cells and vascular smooth muscle cells (SMCs). The differentiation of ECs and SMCs from mesodermal cells was promoted by VEGF and PDGF-BB, respectively (35). Therefore, different small molecules and cytokines can be selected and combined to generate efficient mesodermal progenitor cells for ECs. Lam et al. reported that CHIR99021 effectively induced hPSCs to differentiate into BRACHY^+^ cells expressing mesoderm-specific markers compared with WNT3a treatment (36). Another study also confirmed that treating hiPSCs with CHIR99021 alone at different concentrations and without cytokine support could induce differentiation into BRACHY^+^ mesodermal cells. These cells could further generate CD34^+^/CD31^+^ populations of up to 25% in endothelial differentiation media (37). These studies suggested that efficient mesoderm induction is essential for further EC differentiation.

**Table 1:** Summary of the PSC-derived endothelial cell differentiation protocols using biochemical factors.

Cell sources	Final cell type	Key factors	Duration of differentiation	Functional analysis	Differentiation efficiency	References
iPSCs	ECs	M: 6 μM, 2 μM CHIR EC I: 50 ng/ml VEGF EC II: 30 ng/ml VEGF	7 days	qPCR: CD31, VE-Cad, vWF, VEGFR2 IF: CD31, vWF, Ac-LDL Tube formation	25% for CD31 by FACS	(29)
Keratinocyte-derived hiPSCs	ECs	M: 12 μM CHIR + 30 ng/ml BMP4 3D EC I: 2 μM forskolin +100 ng/ml VEGFA 2D EC II: 100 mg/ml VEGFA +100 ng/ml FGF2	10 days	IF: CD31, VE-Cad, CD146, CD34) Tube formation	35%–50% for CD31 by FACS	(26)
H9-hESCs, CBIA-3, -7, -19, -37, -50, -58 hiPSCs	ECs	PS: 3 μM CP21R7 + 25 ng/ml BMP4 + 50 ng/ml FGF2 M: 25 ng/ml BMP4 + 50 ng/ml FGF2 EC I: 200 ng/ml VEGF +10 μM DAPT +2 μM Forskolin	5 days	Tube formation Dil-Ac-LDL	85%–94% for CD31, CD34, CD144 by FACS	(25)
hiPSCs	Vascular ECs	M: 8 μM CHIR + 25 ng/ml BMP4 EC I: 200 ng/ml VEGF +2 μM Forskolin	7 days	IF: CD31, VEGFR2, VE-Cad, vWF Tube formation	50% for CD31^+^/CD144^+^ by MACS	(30)
hESCs	ECs	M: 6 μM CHIR + 10 ng/ml Activin A + 20 ng/ml BMP4 EC I: 50 ng/ml VEGF + 10 ng/ml bFGF + 20 ng/ml BMP4 + 10 μM DAPT EC II: 50 ng/ml VEGF + 10 ng/ml bFGF + 10 μM SB431542	11 or 15 days	IF: CD31, VE-Cad Dil-Ac-LDL Tube formation	95%–97% for CD31, CD34, VEGFR2 by FACS	(16)
H1-, H9-hESCs	Arterial ECs, Venous ECs	PS: 5 μM CHIR M: 50 ng/ml bFGF EC I: 25 ng/ml BMP4 + 50 ng/ml VEGF	5 days	qPCR: CD34, VEGFR2, CD31, VE-Cad, vWF, eNOS, Tie2 IF: CD31, VE-Cad, vWF, Dil-Ac-LDL Tube formation	85% for CD31^+^/CD34^+^ by FACS	(24)

PS, primitive streak stage; M, mesoderm stage; EC I&II, EC specification I&II; Ac-LDL, acetylated low density lipoprotein; BMP, bone morphogenetic protein; ECs, endothelial cells; eNOS, endothelial nitric oxide synthase; ESCs, embryonic stem cells; FACS, fluorescence-activated cell sorting; FGF, fibroblast growth factor; hESCs, human ESCs; hiPSCs, human induced pluripotent stem cells; MACS, magnetic activated cell sorting; PSCs, pluripotent stem cells; qPCR, quantitative PCR; VE-Cad, vascular endothelial cadherin; VEGF, vascular endothelial factor; VEGFR, VEGF receptor; vWF, von Willebrand factor.

### 2.2. Vascular specification from mesodermal cells

#### 2.2.1. 2D-monolayer system for endothelial differentiation

The use of a 2D monolayer system for endothelial cell differentiation has been extensively studied (24, 29, 37). In a 2D system, iPSCs are seeded on a flat surface, like a tissue culture plate without a feeder layer, and then differentiated into endothelial cells utilizing several growth factors and cytokines (Table 1). This method is easy to use and reproducible for EC differentiation (38). Vascular endothelial growth factor (VEGF) is a critical element controlling EC phenotype specification (39). Through the stimulation of VEGF-mediated downstream signaling pathways, such as the MAPK and PI3K pathways, VEGF can enhance the differentiation of endothelial cells from stem cells (40–42). Several studies have reported efficient induction of PSCs-derived endothelial differentiation by adding VEGF (35, 43, 44). VEGF is a key factor that can promote the differentiation of PSCs-derived endothelial cells but its effect can be enhanced when combined with other growth factors. Sriram et al. demonstrated that the differentiation efficiency of endothelial cells by VEGF and bone morphogenetic protein 4 (BMP4) in mesodermal-committed cells. BMP4, a member of the TGF-β superfamily, is important in directing human stem cells to a mesodermal lineage and facilitating the generation of immature ECs (45, 46). Fluorescence-activated cell sorting (FACS) analysis indicated that BMP4 alone had a minimal effect on the population of endothelial progenitors. In contrast, when treated with VEGF alone, over 50% of the population differentiated over time (by day 5). Counterintuitively, differentiation into CD34^+^/CD31^+^ endothelial progenitor cells (EPCs) was more than 90% successful at day 5 when BMP4 and VEGF were used together (24). Harding et al. also reported the differentiation efficiency of endothelial cells by a combination of various growth factors. They elevated the expression of EC-related genes through the addition of bFGF as well as the combination of VEGF and BMP4 (40). Takeshi et al. reported the direction of differentiation from VEGFR2^+^ mesoderm cells to EC using stimulation with VEGF and cyclic adenosine monophosphate (cAMP) (17). In addition, forskolin, the cAMP pathway activator, was used to promote endothelial commitment during differentiation to EC (25, 26, 30). Therefore, exposure to VEGF is essential for endothelial differentiation from cells of the early mesodermal lineage and its efficiency can be greatly increased by a synergistic combination of cytokines.

Although the 2D-based EC differentiation methods by treatment of growth factors are considered effective ways, the iPSC-ECs can exhibit heterogeneity (19). The concept of EC heterogeneity is widely recognized, as ECs exhibit remarkable diversity in both their functional and gene expression profiles (19, 47, 48). This heterogeneity of ECs and differentiation fate is further regulated by VEGF exposure. High concentrations of VEGF (50 ng/ml) induced the specification of ECs into the arterial subtype, whereas lower concentrations (10 ng/ml) promoted venous specification (19). Another study also supported this tendency, hESC-endothelial progenitors differentiated into arterial and venous subtypes under serum-free culture conditions containing EGF and bFGF, with and without VEGF, respectively. Compared to hiPSC-ECs treated with a high concentration of VEGF throughout the differentiation process, the venous markers, CoupTFII and EphB4, were increased under low VEGF conditions. The number of positive populations of the vein markers NRP2 and EPH-B4 increased in the absence of VEGF. With the addition of 10 ng/ml VEGF, arterial specification of endothelial progenitors exceeded 97%, and the relative mRNA expression levels of arterial and venous-associated markers were regulated by VEGF (24). This highlights that the presence of VEGF also plays a crucial role in arterial and venous EC differentiation from hESC-derived CD34^+^/CD31^+^ EPCs. Therefore, VEGF signaling plays a considerable role in vascular specification, arterial and venous selective differentiation, and maintenance of EC properties of PSC-derived mesodermal/endothelial progenitors. Thus, future efforts will be needed to develop methods to derive iPSCs-ECs with specific origins and functions required in the production of appropriate ECs for the treatment of cardiovascular diseases.

#### 2.2.2. 3D-system for endothelial differentiation

The EB formation approach for generating endothelial cells from stem cells includes plating the stem cells as aggregates and allowing them to differentiate spontaneously in a manner like embryonic development that can progress to the three germ layer (49). While this method results in a heterogeneous population of aggregated cells, it can also lead to low efficiency in generating endothelial cells due to the difficulty in controlling the differentiation process and ensuring that the appropriate signaling pathways are activated. Levenberg et al. first described the approach of EB-derived endothelial cell differentiation. In this method, hESCs were cultured as aggregates in suspension, and toward the endothelial cells, but exhibited very low efficiency of EC generation (approximately 2%) (20). Another method for improving the efficiency of EC differentiation using EBs is continuous addition with VEGF-A, which is known to increase the typically low yield of ECs obtained from EBs (39). Recently, Hamad et al. have established a high-efficiency EC differentiation protocol in a 2D culture system using a high concentration of VEGF and other growth factors. Subsequently, they applied this protocol to a differentiation method starting from 3D EB formation and showed improved EC differentiation yield, compared to previous studies (44). The EB-derived ECs exhibited typical endothelial cell markers such as CD31, VE-cadherin, and von Willebrand factor. In order to use iPSCs-EC as a cell therapy product, a 3D bioreactor suspension system is a promising approach for mass production, and the 3D EB-based EC differentiation method is an inevitable approach and requires further investigation to optimize its efficiency.

### 2.3. Endothelial cell maturation

The appropriate culture conditions used for the maturation of endothelial cells differentiated from iPSCs can be optimized to promote endothelial cell expansion and functional properties after vascular specification. Although VEGF plays a vital role in EC specification, Sriram et al. exposed EPCs (CD34^+^/CD31^+^) to different concentrations of VEGF ranging from 0 to 100 ng/ml and observed that they became apoptotic at high VEGF concentrations. The hESCs-derived CD34^+^/CD31^+^ cells stably maintained EC-specific surface expression for serial passages in the presence of low concentrations of VEGF (24). These results indicated that the optimal concentration of VEGF for EC expansion should be considered after vascular specification.

SB431542 is a small molecule inhibitor that specifically targets the TGF-β signaling pathway (50). Inhibiting TGF-β signaling by SB431542 can enhance the endothelial cell specification and maintenance (51–53). James et al. found that TGF-β inhibition by SB431542 promotes EC specification and expansion of ECs in homogeneous populations, which exhibited high transcriptional signature of typical endothelial cells (51). Treating SB431542 after EC differentiation allows the promotion of endothelial proliferation and prevents senescence during passage (30). The addition of SB431542 for EC differentiation may cause distinct effects depending on which stage of EC differentiation is applied. The use of SB431542 at the early stage of EC differentiation led to a decrease in the expression of mesoderm-specific genes, whereas the addition of SB431542 at the later stage of differentiation increased the expression of endothelial-specific genes (53). These results suggest that inhibition of the TGF-β signaling pathway in the early stage negatively affects the generation of mesodermal cells but has a positive impact on vascular specification and EC maturation.

### 2.4. Vascularization of hPSC-EC

Endothelial cells and mural cells, including pericytes and vascular smooth muscle cells, are essential for vascular development. To evaluate the angiogenic properties of hPSC-EC, Orlova et al. established the monolayer differentiation methods to derive ECs and pericytes from hPSCs and 2D coculture systems consisting of hiPSC-Ecs and pericytes on gelatin-coated dishes to model vascular plexus (31, 43). Briefly, hPSC-Ecs first adhere to the substrate and form EC islands surrounded by pericytes that remodel into vascular-like structures. Kurokawa et al. investigated the ability of the CDH5-iPS-ECs to form 3D vascular networks by co-culturing EC with fibroblast in microfluidic devices (54). The iPS-ECs undergo vacuole formation and aggregate into tube-like structures. The microvessel network connected to the top and bottom microfluidic lines and was perfusable. Recently, several studies reported the development of an organ-on-a-chip model to recapitulate a pathophysiological microenvironment. Campisi et al. established a human blood-brain barrier (BBB) microvascular network model with hPSC-EC, brain pericytes, and astrocytes in a fibrin gel (55). The BBB model exhibited perfusable and selective microvasculature, with permeability lower than that regarded conventional *in vitro* models. Wimmer et al. attempted to generate self-organizing 3D human blood vessel organoids from hPSC that exhibited vascular network formation in the collagen I–Matrigel matrix (56). Vessel organoids differentiated into CD31^+^ endothelial networks and PDGFR- β^+^ pericytes. That also showed sprouting vascular networks at the periphery and prototypical tip cell morphology. In addition, the transplantation of blood vessel organoids into immunocompromised mice formed a stable human vasculature. Therefore, the development of vascular modeling and organoid using hPSC-EC can be applied to understanding vascular disease and vascular regeneration.

## 3. Influence of ECM cues on EC differentiation

### 3.1. Extracellular matrix as a regulator for stem cell behavior

The microenvironment surrounding ECs is defined as the “vascular endothelial niche”. ECs are located in the basement membrane (57), whose components are proteoglycans and proteins such as collagen IV (Col-IV), laminin, and fibronectin (58, 59). The ECM of the vascular niche plays a critical role in vascular tissue engineering by providing both a physical scaffolding for adhesive support and a 3D environment by transmitting biochemical and mechanical signals that control cellular behavior. The stiffness of the ECM is one of the most critical factors in the microenvironment and plays a vital role as an inducer in the behavior of different cell types (14, 15, 60). Various hydrogels, such as polydimethylsiloxane (PDMS), polyacrylamide (PAA), collagen, and gelatin methacryloyl (GelMA), can be used to mimic tissue-specific stiffness (61, 62). Single-stiffness and stiffness gradient hydrogels were simulated to study the mechanotransduction and behaviors of stem cells that are mechanosensitive to matrix stiffness (14, 63, 64). Stem cells can mechanosense the rigidity of the matrix; information about the rigidity is transmitted to the nucleus along the cytoskeleton, and, Yes-associated protein (YAP), Lamin A, and MRTFA, which are mechanosensitive proteins; these mechanosensitive proteins react according to the stiffness of the matrix and generate biochemical signals that ultimately determine stem cell behaviors such as migration, proliferation, functions, and differentiation fate of stem cells (63–66). For example, a soft matrix promotes adipogenesis and a stiff matrix promotes osteogenesis (67). The results indicated that stem cell mechanosensitive proteins are sensitively regulated depending on the substrate stiffness (soft to stiff) and even influence their differentiation fate. Therefore, using an appropriate matrix stiffness is vital to differentiate stem cells into the endothelial lineage (Figure 1B).

### 3.2. Mechanical properties of the vascular environment

**Figure 2:**
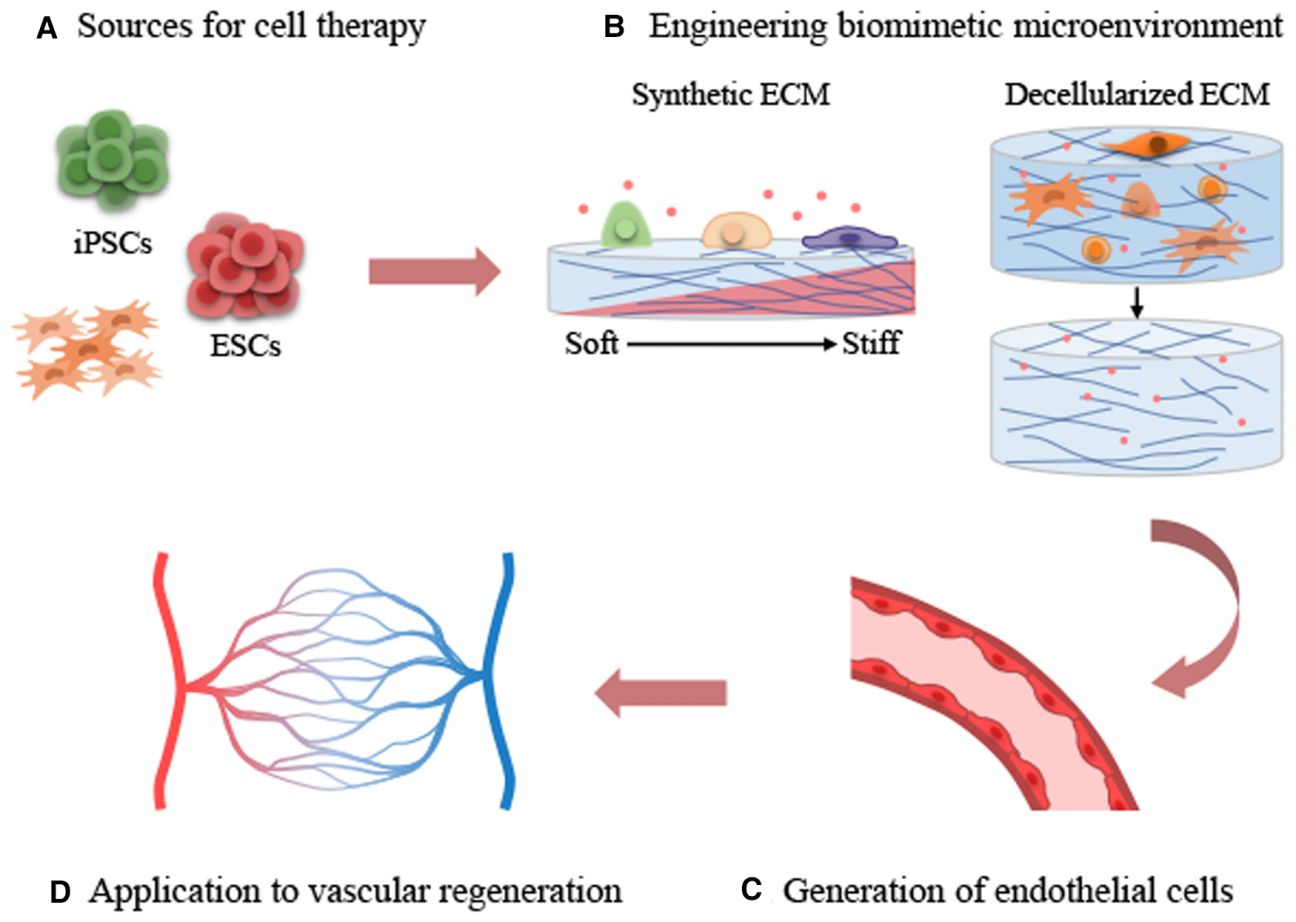
Applications of extracellular matrix cues on stem cell-derived endothelial differentiation and vascular regeneration. (**A**) Sources for cell therapy. (**B**) Engineering biomimetic environment. (**C**) Generation of endothelial cells. (**D**) Application to vascular regeneration.

Developing tissue constructs that incorporate the mechanical properties of natural tissues is essential to provide proper mechanical cues to cells. Substrate stiffness is a key factor among the mechanical properties of ECM, and it is important to understand the stiffness of natural tissues. To this end, atomic force microscopy (AFM) is a valuable tool for measuring the stiffness of tissues and living cells (68, 69). The elastic modulus (*E*) of the vascular environment varies by species and tissue, as shown by AFM. For example, the stiffness values of murine femoral artery and thoracic aorta were 3.2 and 4.3 kPa, respectively, as revealed by AFM measurements (70). According to reported AFM indentation measurements, the mean elasticity of the tunica adventitia layer of porcine pulmonary arteries and aorta were 128.6 and 25.8 kPa, respectively (71). In contrast, measurements of the intima of bovine carotid and the media layers of porcine carotid arteries yielded mean stiffnesses of 2.5 and 5.7 kPa, respectively (72, 73). The reported elastic modulus of venous tissue is approximately 3–50 kPa, which is in between the stiffness of the epithelium and cartilage (11, 74, 75). Unlike veins, arteries are composed of a high density of Col-I and are surrounded by several layers of SMCs and connective tissue, allowing them to withstand high blood pressure (74, 76). Therefore, arterial tissues have higher stiffness values than veins within a range of elastic moduli 50–150 kPa (71, 75, 77, 78). Finally, exploring the mechanical properties of the vascular niche and highlighting the significance of engineering biomimetic environments for stem cell differentiation for application in vascular regeneration is necessitated (Figure 2).

### 3.3. Effects of substrate stiffness on endothelial differentiation

The endothelial commitment of stem cells can be regulated by substrate stiffness (Table 2). Smith et al. demonstrated that compliant substrates could guide the endothelial commitment of hPSCs. The authors used physiologically soft (3 kPa) and stiff (1.7 MPa) matrices fabricated by PDMS substrates to compare the traditional TCP environment (3 GPa) for culturing and EC differentiation from hPSCs. They found that hPSCs cultured on the soft matrix enhanced differentiation toward endothelial cells through stiffness-mediated mesodermal induction, with robustly improved EC marker expression including VE-cadherin, CD31, vWF, and eNOS (82). The differentiation of EPCs into arterial or venous ECs *in vitro* is suggested to be influenced by variations in substrate stiffness. Xue et al. prepared PDMS-based matrices with stiffness physiologically related to venous (7 kPa) and arterial (128 kPa) tissue for inducing differentiation of mouse bone marrow-derived EPCs on each matrix. When the correct substrate stiffness was applied, the expression of EphrinB4, a venous endothelial marker, was upregulated on a soft (venous) substrate, whereas stiff (arterial) substrates increased the expression of EphrinB2, an arterial endothelial marker (11). These studies demonstrated the important role of substrate stiffness in regulating endothelial differentiation and functionality, emphasizing the necessity of using substrates that mimic the physiological conditions of the vascular niche in tissue engineering applications.

**Table 2:** Summary of the mechanical cue-based endothelial cell differentiation protocols.

Cell sources	Final cell type	Substrates	Key factors	Differentiation time	Functional analysis	Differentiation efficiency	Reference
PGP1-iPSCs	Heart valve endothelial cells (VECs)	PDMS coated with Col-IV Soft: 40 kPa, Medium: 3.2 MPa, Stiff: 2.8 GPa	M: 25 ng/ml WNT-3a + 10 μM CHIR + 10 ng/ml BMP4 CPC: 50 ng/ml BMP4 + 10 μM CHIR + 20 ng/ml bFGF VEC: 100 ng/ml VEGF + 50 ng/ml bFGF	10 days	qPCR: VE-Cad, CD31, NFATC1, vWF IF: CD31, VE-Cad, NOS3, NFATC1 mRNA seq analysis	43.6%–60.4% for CD31 by FACS	(12)
Murine R1-ESCs, A3-ESCs	VPCs	PAA gel: 10, 40 kPa Coated with 50 μg/ml Fibronectin	VPC: 5 ng/ml BMP4 + 30 or 20 ng/ml VEGF − TCP (50 μg/ml Fibronectin) EC I: 30 ng/ml VEGF + 5 ng/ml BMP4 or 10 ng/ml VEGF + 10 ng/ml bFGF	14 days	IF: PECAM-1, CNN1 mRNA seq analysis	ㅡ	(10)
hiPSCs (WISCi004-A, BIHi004-A, BCRTi005-A)	ECs	Human kidney-dECM HSPG(−)/HSPG(−), VEGF(+)/HBGF(−)/HBGF(−), VEGF(+)	M: 1.5 μM CHIR + 30 ng/ml BMP4 + 25 ng/ml Activin A + 50 ng/ml VEGF EC I: 50 ng/ml VEGF + 10 μM SB431542	10 days	IF: CD31, CD144	ㅡ	(79)
Mouse BM-derived EPCs	Arterial ECs, Venous ECs	PDMS Venous: 7 kPa, Arterial: 128 kPa	EC: 10 ng/ml VEGF + 3 ng/ml bFGF	15 days	qPCR, WB: ephrinB2, EphB4 IF: ephrinB2, EphB4	ㅡ	(11)
iPSCs (HUF5, DOX1), H1-ESCs	ECs	ECM-coated Microarray Col-IV, Fibronectin, Laminin, Gelatin, Heparan sulfate, Matrigel ECM concentration: 0.5 mg/ml	M: 50 ng/ml VEGFA + 50 ng/ml BMP4 EC I: 50 ng/ml VEGFA	5 days	qPCR: CD31, VE-Cad, FLK1 IF: CD31	26.6%–48.3% for CD31/VE-Cad^+^ by FACS	(80)
Human umbilical cord blood-derived EPCs	ECs	Porcine vascular tissue-dECM	EC I: EGM2 MV SingleQuot kit	7 days	qPCR: CD31, VE-Cad, vWF IF: CD31	ㅡ	(81)

PS, primitive streak stage; M, mesoderm stage; EC I, EC specification I; CPC, cardiac progenitor cell; VECs, valve endothelial cells; bFGF, basic fibroblast growth factor; BM, bone marrow; Col-IV, collagen IV; dECM, decellularized extracellular matrix; ECM, extracellular matrix; EPCs, endothelial progenitor cells; PDMS, polydimethylsiloxane; TCP, tissue culture plate; VPCs, vascular progenitor cells.

Flk-1 is a well-known marker of vascular progenitor cells (VPCs), which can differentiate into ECs and SMCs (83). The formation of these Flk-1^+^ cells can be influenced by the substrate stiffness to which the cells are exposed. For instance, when comparing the differentiation ratio of mesenchymal stem cells (MSCs) in stiff (8–15 kPa) and soft (2–5 kPa) 3D nanofiber substrates, 95% of MSCs exhibited Flk-1 endothelial markers on soft substrates within 24 h. However, only 20% of MSCs presented Flk-1 markers on stiff matrices (84). Wong et al. demonstrated that the differentiation of ECs and SMCs from Flk-1 cells is related to substrate stiffness. They prepared soft (10 kPa), medium (40 kPa), and stiff (>100 kPa, TCP) hydrogels with a PAA substrate that can mimic most physiological stiffness levels. Flk-1^+^ cells differentiated from murine ESCs adhered to each matrix for EC differentiation (10). After ESCs differentiated into VPCs (Flk-1^+^ cells), the differentiation potential of ECs and vascular SMCs varied depending on the rigidity of the matrix. The rigid matrix directed VPCs to CNN1^+^ SMCs, whereas softer hydrogels resulted in more CD31^+^ ECs. Beyond the effects of the 2D matrix on understanding PSCs-derived EC differentiation, the 3D hydrogels can enhance the differentiation efficiency of ECs. Zhang et al. found that the 3D environment by fibrin scaffold elevated the efficiency of EC differentiation and quality of hiPSCs-ECs compared to the traditional 2D culture system. This approach suggested that providing the physical 3D surface tension by controlling the spatiotemporal environment is important for enhancing EC differentiation and the function of ECs (85). Although the stem cells had the same starting point, their differentiation fate changed depending on the substrate stiffness to which they were exposed. Thus, the stiffness-dependent lineage commitment suggests that the optimal substrate stiffness parameter is vital for the efficient generation of ECs from PSCs.

### 3.4. Effect of extracellular matrix composition on endothelial differentiation

Recent studies in mechanobiology have focused on matrix stiffness, a mechanical property of the ECM, and have confirmed that it is a critical factor in determining numerous cellular behaviors (14, 15). However, not only the substrate stiffness of the ECM but also the type and density of ECM ligands can affect stem cell mechanotransduction beyond the effect of matrix stiffness. Notably, specific ECM types and their densities can even reverse the effect of substrate stiffness. Stanton et al. demonstrated that YAP-mediated mechanotransduction of stem cells is regulated by ECM types such as fibronectin, Col-I, Col-IV, and laminin with different ECM ligand densities (86). These results highlight the importance of biochemical ligand type and density in designing mechanotransduction experiments and applying the optimal ECM components for EC differentiation studies.

Various types of ECM materials have been used for PSCs-derived endothelial differentiation, including Matrigel, fibronectin, and laminin (16, 25, 44). Matrigel, a commercially available ECM product derived from Engelbreth-Holm-Swarm mouse sarcomas, is widely used for supporting growth and differentiation of various cell types, including endothelial cells (24, 87, 88). However, there are several drawbacks to using Matrigel for EC differentiation. Matrigel shows batch-to-batch variation and contains many undefined growth factors, which can interfere with the reproducibility of experiments (89, 90). Therefore, alternative ECM materials such as fibronectin and laminin have been explored for PSCs-derived EC differentiation. Siram et al. established a protocol for efficient differentiation of hESCs into ECs under feeder- and serum-free conditions, using fibronectin as an alternative ECM to Matrigel. They found that culturing hESCs on the fibronectin-coated surfaces can maintain the pluripotent status and induce differentiation of ECs (24). Nguyen et al. demonstrated the ability of laminin to support the differentiation of hESCs into functional endothelial progenitor cells under chemically defined, xeno-free conditions (16). These highlight the potential of using alternative ECM materials for efficient EC differentiation, which could aid cell-based therapies for vascular disease, and emphasizes the importance of utilizing defined ECMs for reliable and reproducible differentiation protocols.

A combinatorial ECM microarray platform was used for PSC-derived EC differentiation to understand the influence of the microenvironmental factors regulating their differentiation. ECM microarrays consist of multi-component combinations of the ECM proteins, including fibronectin, Col-IV, laminin, gelatin, heparin sulfate, and Matrigel to mimic the environment of the basement membrane where ECs reside (80). Human iPSC or ESC lines were differentiated into ECs on an ECM microarray, and the relative fluorescence intensity of CD31 was compared under each condition. Compared with other ECM components, combinatorial ECMs (CHL: Col-IV + heparan sulfate + laminin, and CGH: Col-IV + gelatin + heparan sulfate) could promote endothelial differentiation by increasing the expression of CD31 at the protein and transcript levels (80). These results suggest that optimal ECM components and combinations are essential for efficiently producing ECs from PSCs.

### 3.5. Decellularized extracellular matrix as differentiation inducers

Decellularized ECM (dECM) is a commonly used natural biomaterial that can be obtained by removing cells from tissues or cells with detergents and can be derived from various types of organs or tissues (91, 92). The dECM preserves the characteristics of the natural ECM and provides a structural scaffold, biomechanical properties, soluble components, native proteins, and cell adhesion ligands (93, 94). Recent research on tissue- and organ-derived ECM emphasizes the need for tissue specificity to maintain the function and phenotype of native cells (94–96). Therefore, vascular tissue-derived ECM (VdECM) may be an attractive biomaterial to recapitulate the vascular niche and enhance EC differentiation and cell function. VdECM and Col-I gels were used to assess cell proliferation, viability, and differentiation to evaluate the performance of encapsulated EPCs. The proliferation ability of live cells in VdECM significantly exceeded that of Col-I gel, as confirmed by cell viability and proliferation assays. Compared with Col-I gel, gene expression of typical endothelial markers, including CD31, CD144, and von Willebrand factor, was significantly increased in VdECM. Moreover, mature CD31^+^ ECs formed microvascular-like structures, which is a distinguishing feature in contrast to the simply elongated morphology of ECs in Col-I gel (81). Ullah et al. demonstrated that the VEGF-supplemented dECM is an instructive material for effective differentiation of hiPSCs into ECs *in vitro* and even in the absence of other inductive factors in culture medium. The VEGF-enriched dECM provides a functional niche for the selective promotion of cell attachment, survival, and differentiation (79). This study provides important insight into the role of specific growth factors in dECM that promote EC differentiation instead of conventional growth factor-supplemented media. By identifying and supplementing specific dECM factors, it may be possible to enhance the differentiation efficacy and functionality of PSCs-EC for regenerative applications. These results suggest that the natural properties of dECM can be used as an efficient tool for vascular EC differentiation.

## 4. Conclusion

In recent years, different types of stem cells and various approaches have been developed to generate ECs using chemical treatments. Most of the research is currently performed with 2D monolayer-based protocols to guide mesoderm induction and endothelial specification. The appropriate type and concentration of growth factors for differentiation stages are essential for effective EC generation. Many studies have been performed on plates coated with Matrigel or other ECM components (laminin, collagen, and fibronectin) and treated with growth factors. However, stem cells can “mechanosense” their ECM stimuli and convert them into biochemical signaling pathways. Synthetic matrix and tissue-specific dECM can be used to recapitulate the vascular microenvironment by applying this concept to EC differentiation. Modulating the stiffness and components of the ECM is critical in determining the differentiation fate of arterial and venous ECs. Therefore, investigating mechanical factors related to the vascular microenvironment and their application to EC generation is necessary to find novel strategies and directions for treating cardiovascular diseases.
